# Oligodendrocytes in the aging brain

**DOI:** 10.1042/NS20210008

**Published:** 2021-07-06

**Authors:** Eleanor Catherine Sams

**Affiliations:** Blizard Institute, Barts and The London School of Medicine and Dentistry Centre for Neuroscience, Surgery and Trauma, Blizard Institute, 4 Newark Street, Whitechapel E1 2AT, London

**Keywords:** aging, cognition, myelin, oligodendrocytes, senescence

## Abstract

More than half of the human brain volume is made up of white matter: regions where axons are coated in myelin, which primarily functions to increase the conduction speed of axon potentials. White matter volume significantly decreases with age, correlating with cognitive decline. Much research in the field of non-pathological brain aging mechanisms has taken a neuron-centric approach, with relatively little attention paid to other neural cells. This review discusses white matter changes, with focus on oligodendrocyte lineage cells and their ability to produce and maintain myelin to support normal brain homoeostasis. Improved understanding of intrinsic cellular changes, general senescence mechanisms, intercellular interactions and alterations in extracellular environment which occur with aging and impact oligodendrocyte cells is paramount. This may lead to strategies to support oligodendrocytes in aging, for example by supporting myelin synthesis, protecting against oxidative stress and promoting the rejuvenation of the intrinsic regenerative potential of progenitor cells. Ultimately, this will enable the protection of white matter integrity thus protecting cognitive function into the later years of life.

## Introduction

Continuing progress in science and healthcare has led to an increased life-expectancy and an aging population in many parts of the world. The rate of population aging is now accelerating rapidly across countries of all income status and The World Health Organization predicts that by 2050, 22% of the world’s population will be in the over-60 age group. Along with these advancements in public health comes the challenge of coping with rising socioeconomic pressure from age-related disabilities, such as cognitive decline.

Longitudinal studies show that cognitive decline across the lifespan largely affects working executive memory and the slowing of processing speed [1,2], which can make daily tasks challenging by affecting sensory, cognitive and behavioural processes. Although the exact mechanisms of cognitive decline are not yet known, it is understood that progressive breakdown of the intricate communication between neurons and glial cells, reduced efficacy of action potential conduction and processes such as neuroinflammation lead to a non-autonomous and gradual loss of cognitive function [3]. White matter tracts functionally connect various areas of the central nervous system (CNS), and are predominantly populated by myelinated axons. A focus on the importance of white matter in aging was introduced and intensely researched by Bartzokis and colleagues [4–6]. This has led to a growing field of interest and understanding of brain aging as a network deterioration, such that the loss of myelination in white matter tracts which connect cortical regions underlies the loss of cognitive functions which rely on this network connectivity and efficient neuronal transmission. Non-human primate work has found direct links between reduced myelination index, measured using diffusion tensor imaging (DTI), of specific corticocortical and corticobasal tracts and cognitive performance in normal aging [7,8].

In the CNS, myelin is provided by terminally differentiated cells of the oligodendrocyte lineage, which hereafter will be referred to as mature oligodendrocytes. Developmental myelination of the CNS takes place largely within the first 2 years of life, but white matter volume increases up until around mid-life as new axonal projections become myelinated. Adult myelination is highly plastic, modifiable by experience, and seems to have important roles in learning and memory and normal cognitive function [9]. Oligodendrocytes are derived from specific neural progenitor cells; oligodendrocyte progenitor cells (OPCs), also known as neuron-glial antigen 2 (NG2)-positive glia. OPCs populate the CNS, and proliferate throughout life to self-renew, and differentiate to provide a continuous source of new mature oligodendrocytes. A variety of less well-characterised roles of OPCs in the adult brain are also emerging, such as roles in neuronal-glial signalling, electrical activity, phagocytosis [10], and stem cell-like behaviour [11]. Specific developmental phenotypes from progenitor cell to terminally differentiated cell can be traced through the expression of oligodendrocyte-specific proteins, for example the myelin-specific proteins such as myelin basic protein (MBP) and myelin oligodendrocyte glycoprotein (MOG) are expressed only by mature oligodendrocytes, whereas the proteoglycan NG2 is expressed at the OPC stage and then down-regulated [12,13].

**Figure 1:**
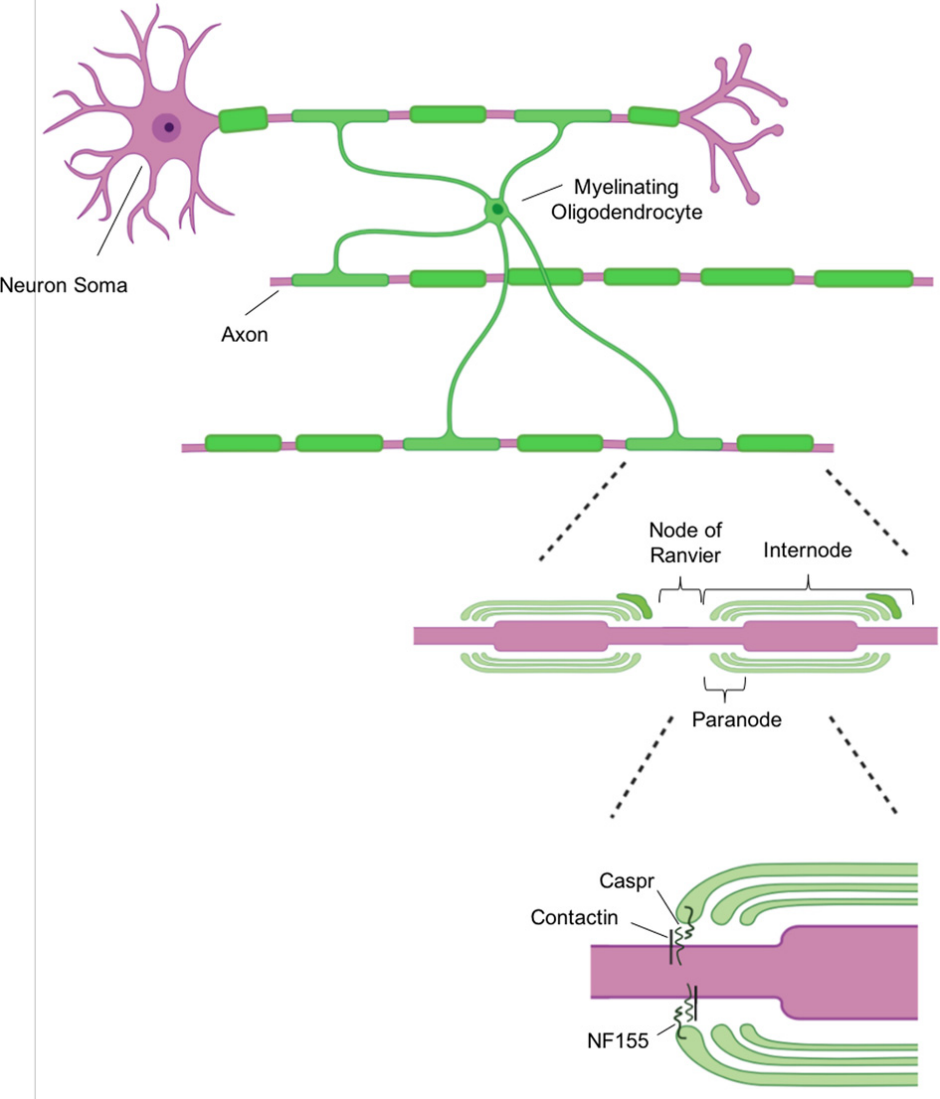
Organisation of myelin internodes on CNS axons Mature oligodendrocytes extend multiple processes to concentrically wrap axons in myelin membrane. Myelination is organised in internodes. Nodes of Ranvier are exposed areas of axon, where ion channels are clustered, which promotes saltatory conduction. Specialised proteins, neurofascin 155 (NF155), contactin and caspr, physically attach the internode to the axon, at the distal regions of each internode, known as the paranodes.

Myelin is a lipid-rich membrane structure, which wraps concentrically around axons, forming patterns of myelinated axonal lengths: internodes, separated by unmyelinated nodes of Ranvier (Figure 1). This organisation allows the clustering of ion channels at the nodes of Ranvier, to increase propagation speed of electrical signalling along the length of an axon by saltatory conduction [14]. As well as increasing conduction speed, myelin has numerous emerging roles in metabolic support of axons. For example, lactate and pyruvate transfer via monocarboxylate transporters provides a metabolic substrate for axons [15–19]. The myelin internode is physically attached to the axon at the paranodes, the distal ends of each internode, by specialised contact proteins neurofascin 155, caspr and contactin (Figure 1). Axonal signals orchestrate trafficking of myelin proteins within oligodendrocytes and OPCs also receive synaptic connections from neurons, which regulates their transition from a proliferative state towards differentiation [20]. Therefore, there is complex bidirectional signalling between neurons and the oligodendrocytes which highlights the significance of oligodendrocytes to overall brain homoeostasis and connectivity.

Here, we focus centrally on mature oligodendrocytes and OPCs within the aging brain environment and consider the impact of intrinsic and extrinsic age-related alterations on the ability of these cells to maintain myelination within CNS white matter. We will first consider the age-related changes that occur within white matter that contribute to a reduction in end-of-life quality. We will then discuss the basis of aging, i.e. what is aging, why does it happen and why do we believe it is both feasible and worthwhile to focus our research efforts into modifying its natural progression? Finally, a review of the current state of knowledge on the age-related changes that occur within cells of the oligodendrocyte lineage, including intrinsic factors such as cellular senescence and extrinsic influences such as interactions with other neural cell types, neuroinflammation, niche environment, and energy and nutrient availability, and the progress that is being made towards effective interventions to protect these cells and promote efficient white matter maintenance in the face of age-related failure.

## White matter degeneration correlates with decreased cognitive function in normal brain aging

It is widely accepted that there is an overall loss in white matter volume with age in non-pathologically aging human brains [21,22]. Early evidence for this came from histochemical studies, where a decrease in Haematoxylin staining intensity was reported in Gennari’s stripe of the striate cortex with increasing age [23]. In the 40 years that have passed since this observation, advancements in medical imaging, specifically DTI have allowed the characterisation of the nature of white matter loss in more detail, through measures of directional water diffusivity [24,25], which can be implemented in longitudinal aging studies [26]. An increase in the DTI ‘relaxation rate’ indicates degeneration of white matter due to increased water content and decreased directionality. This has revealed that there is a peak in total white matter volume at approx. 30 years old, which declines from then after [27–30], with greatest levels of decline in the prefrontal cortex and anterior corpus callosum [21,31–33]. This reflects the anterior-posterior gradient of developmental myelination, such that later-myelinated regions present earlier with age-related decline.

Considering the widespread and specialised roles of myelin, it follows that myelin degradation leads to cognitive decline during ‘normal’ aging, that is in the absence of clinical age-related pathology such as dementia. This is not least as a result of leaving axons exposed and vulnerable to damage, as is well documented in demyelinating conditions such as multiple sclerosis [34]. Longitudinal DTI data show that age-related myelin degeneration largely contributes to loss of cognitive function through disconnection of cortical regions, due to slowed processing speeds [35], which in fact appears to be independent of axonal degeneration [30,36,37]. Processing speed is a key determinant of working memory efficiency [38]. Furthermore, recent studies have demonstrated that toxin-induced demyelination in the hippocampus leads to learning and memory impairments in rodents as a result of decreased neurogenesis [39,40]. Therefore, white matter loss and degeneration may result in age-related cognitive decline via several independent mechanisms. Although this review is focussed on non-pathological brain aging, it is important to note here that age is the main risk factor for age-associated degenerative diseases such as Alzheimer’s disease (AD). There is growing evidence for white matter disruption in these diseases and a broadening of investigation beyond the grey matter pathologies. The chronology of neuronal loss and myelin damage is not yet understood [41]. Therefore, it could be hypothesised that a good understanding of the health of oligodendrocytes in the aging brain and how white matter might be protected in aging is ever more important as a potential prophylactic approach to age-associated disease.

Alongside an overall decline in the volume of white matter in the brain, at the level of the individual internode, myelin shows a degenerative phenotype in the aging brain. Degenerative abnormalities include accumulation of dark cytoplasm [42], formation of myelin balloons [43,44] and piling of paranodal loops [45]. Biochemical changes in the composition of myelin have been reported with age. Human myelin comprises 70% lipid and 30% protein. Analysis of myelin composition in the aging primate brain shows that the lipid content of myelin, especially that of cholesterol, one of the major myelin lipids, shows greater changes than the protein composition, which remains more stable [46]. Furthermore, a recent lipidomic analysis spanning a 12-year period showed that reduced blood levels of membrane lipids, including sphingomyelin was associated with cognitive decline in participants [47]. This suggests that changes in lipid content, rather than protein content, of myelin are largely accountable for myelin changes in cognitive decline. However, it is likely that even subtle changes in myelin composition will contribute to alterations in its numerous functions, through altered protein function as well as extent of axonal insulation due to lipid availability and turnover. It is possible that nutrient delivery is compromised with age, and myelin can be broken down as a ketogenic fuel supply when brain metabolism is compromised [48]. Therefore, ultrastructural changes and fragmentation of myelin may be a result of changing myelin composition due to malnutrition (i.e. metabolic and nutritional deficiencies), oxidative stress or other intrinsic changes that impair the myelinogenic pathways. Age-related degenerative myelin status, including reduced thickness and density of myelin in particular brain regions, namely the prefrontal cortex and regions II and V of the corpus callosum, of non-human primates correlates with decreased measures of cognitive function [49–52].

The loss of white matter volume and the accumulation of myelin abnormalities, which are well defined both in rodents and non-human primates, suggests that oligodendrocytes within the aging brain have a reduced capacity for producing and maintaining healthy myelin sheaths. Furthermore, OPCs fail to rescue this through regenerative *de novo* myelination via differentiation and integration of new oligodendrocytes. This failure is likely due to both intrinsic age-related changes within oligodendrocytes and OPCs, and extrinsic influences from their environment and neighbouring cells. These will be discussed in detail later, but first it is also important to consider what is meant by aging at both the organismal and cellular levels and the rationale behind rejuvenating approaches to age-related disabilities.

## Defining aging and senescence

The lifespan of an organism is defined as the maximum number of years that it is possible to live, while healthspan is used to describe the number of years that can be expected to live free of major disease and disability. As we near the later years of our life, it is accepted and recognised that we will accumulate increasing disability and disease as organs and biological systems become less efficient and begin to fail. Eventually, if not due to any other named disease, every organism will die ‘of old age’. However, the evolutionary significance of this remains unclear. The disposable soma theory of aging states that there is in fact no genetic basis for aging as such, only insufficient programs for survival. Accumulation of random damage with age becomes metabolically too costly to repair, and energy resources are better invested in reproduction, hence evolution allows the eventual degeneration and disposal of the extrinsic soma to protect longevity of genetic transmission [53].

While life expectancy can increase through better management of age-related diseases, and indeed has been extended dramatically in the last century, it is widely agreed that maximum lifespan is largely impervious [54]. Although it should be noted that there are also those that believe that this is not the case and effective therapeutic rejuvenation may lead to a significant increase in possible lifespan. However, the complexities of these arguments are beyond the scope of this review. Here instead we focus on the observation that a particular failure of gerontology research is reflected in the gap between improvement in life expectancy and healthspan, as approx. 16–20% of an individual’s life will now be spent in late-life morbidity [55]. Efforts to reduce late-life morbidity require a detailed understanding of the processes leading to accumulation of age-related damage. Therapeutic strategies to repair this damage before accumulation reaches the threshold of significant disability will protect efficient function of such systems into the later years, extending the proportion of life spent free of disability but within the limits of current life expectancy. Whether or not the actual limit of human lifespan is extendable, or indeed whether this is appropriate or ethical, a healthier and more independent older portion of society is certainly desirable, both from a socioeconomic and personal perspective.

**Figure 2:**
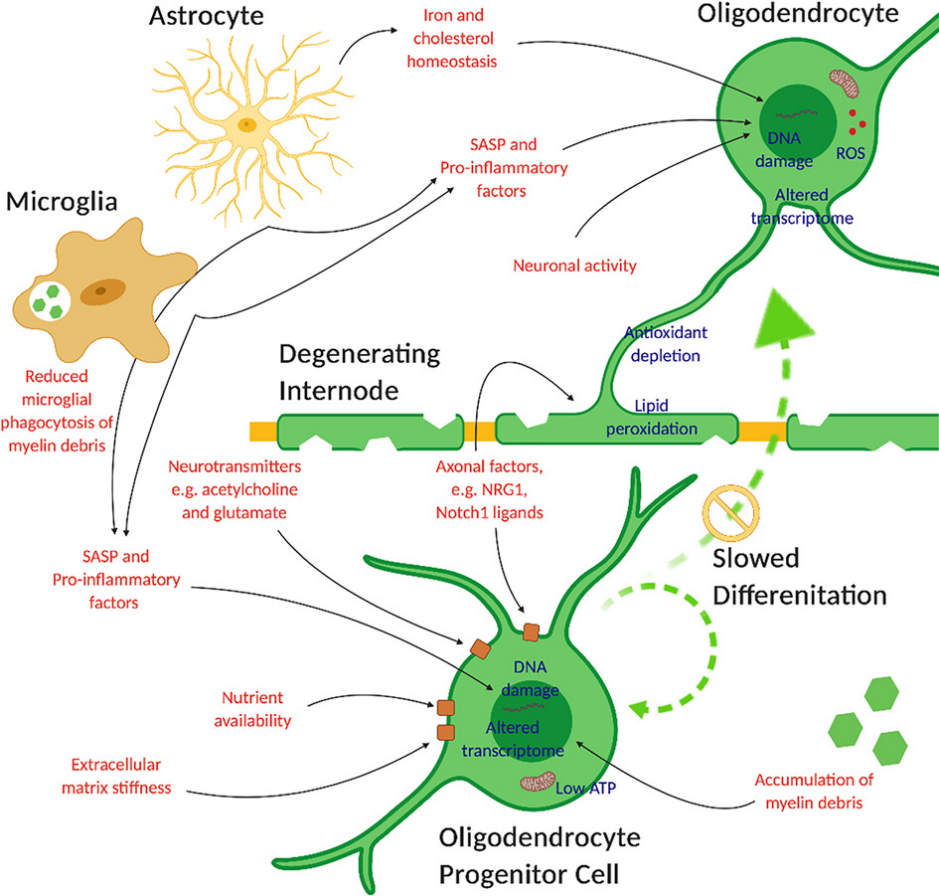
Intrinsic and extrinsic aging factors which influence the ability of OPCs and mature oligodendrocytes to maintain optimal myelination in the aging brain Intrinsic factors (blue) include hallmarks of cellular senescence, such as accumulation of DNA damage, reactive oxygen species (ROS) as well as signs of oxidative stress, altered lipid metabolism, transcriptomes and metabolism. Extrinsic factors (red) include the interactions with other glia, effects of their senescence-associated secretory phenotype (SASP) and pro-inflammatory secretions, altered axonal–glial interactions including axonal factors such as neuregulin 1 (NRG 1), nutrient and energy availability and extracellular matrix properties. This is by no means an exhaustive list but illustrates the complex network of influences acting on cells of the oligodendrocyte lineage in aging.

In order to understand the process of age-related degeneration, focus must be directed towards specific age-related changes in individual cells, which leads to a loss of sufficient physiological capacity to support vital organs and biological systems [56]. In the context of white matter degeneration in aging, a combination of myelin degradation and insufficient myelin maintenance accumulates. As postulated above, by understanding the processes which lead to this failure of repair and maintenance, therapeutic strategies to prevent its advancement with age will prevent the development of associated disability and therefore contribute to an extension of the healthspan. Here we will discuss the current understanding of age-related changes in myelinating oligodendrocytes and their progenitor cells which underlie their failure to maintain adequate myelination and optimal white matter health (Figure 2). These include intrinsic cellular changes such as DNA damage, telomere shortening, cell cycle dysregulation, epigenetic and transcriptional changes, protein and lipid dysfunction, energy impairment and oxidative stress. Extrinsic factors which may be a result of aging and contribute to reduced oligodendrocyte function include the accumulation of cytokines, growth factors, oxygen and nutrient availability, inflammation, and interaction with other cellular, molecular and physical environmental components.

## Mature oligodendrocytes fail to sustain sufficient myelinogenesis due to extrinsic and intrinsic aging events

Mature oligodendrocytes are the terminally differentiated cells of the oligodendrocyte lineage, with a specialised CNS function to produce and maintain the myelin sheaths of myelinated axons. Once integrated, oligodendrocytes are highly stable long-lived cells [57,58]. There is accumulating evidence that these established cells demonstrate intrinsic plasticity via internode remodelling. For example, in response to sustained Erk1 and Erk2 activation, established oligodendrocytes are able to thicken their existing myelin sheaths by addition of new myelin [59]. Furthermore, the internodes can also be lengthened in response to specific ablation of the neighbouring oligodendrocytes [60]. Moreover, a recent study has demonstrated that mature oligodendrocytes can form new processes to repair demyelinated regions at the same time as maintaining their existing extrusions [61]. Remyelinated axons can be identified by the G-ratio of their myelin sheath; the ratio of sheath diameter to axonal diameter, as remyelination consistently produces thinner myelin sheaths than developmental myelination [51,62]. In two separate models, a feline-irradiated food model of demyelination and a non-human primate vitamin B12 deficiency model, electron micrograph images identify oligodendrocyte cells with processes extending to mature myelin sheaths with low G-ratios, as well as sheaths formed via remyelination and categorised as having a high G-ratio [61]. A recent study has further shown, using *in vivo* two-photon imaging, that mature oligodendrocytes in the motor cortex contribute to remyelination following cuprizone-induced demyelination in response to an increase in neuronal activity via implementation of a motor learning task during recovery [63]. Therefore, the mature oligodendrocyte cells themselves display potential for myelin maintenance and remodelling through thickening and lengthening of existing internodes, and establishment of *de novo* internodes. However, in aging, the requirement for myelin repair and remodelling remains unmet. Therefore, it is possible that intrinsic and extrinsic age-related events impair the ability of mature oligodendrocytes to support myelin maintenance in aging by upholding sufficient rates of myelinogenesis, internode remodelling and remyelination.

### Extrinsic influences

#### Cellular senescence in neighbouring glial cells

The term ‘senescence’ is occasionally used to denote aging in general, but here we will use it to refer to ‘cellular senescence’, which is a specific function of cellular aging. This theory of cellular aging states that each dividing cell undergoes a finite number of replications before entering a senescent state which ultimately leads to a termination of cell division. This was first proposed by Hayflick in the 1960s, when it was observed that cells would only divide a finite number of times (40–60 divisions) in culture before permanently exiting the cell cycle and entering what he termed a ‘senescent state’ [64–66]. While the above describes cellular senescence as an exhaustion of possible divisions, ‘premature cellular senescence’ (also termed ‘stress-induced cellular senescence’) has also been described, whereby a cell takes on a senescent phenotype due to other stressors, including epigenetic alterations [67], oxidative damage [68–70] and DNA damage [71,72]. Senescent cells have a senescence-associated secretory phenotype (‘SASP’). SASP is a complex combination of pro-inflammatory chemokines and other inflammatory factors such as migration inhibitory factor (MIF), interleukins, proteins and growth factors such as angiogenin, as well as receptors, including epidermal growth factor receptor (EGF-R) and factors such as nitric oxide and fibronectin [73,74].

The physiological role of cellular senescence is one of protection and regeneration; a means of preventing tumour generation and flagging dysfunctional cells for immune system clearance [75,76]. However, these beneficial effects depend on efficient clearance, and therefore only transient exposure of surrounding cells to senescent cells and their SASP. Many factors can contribute to the development of replicative or stress-induced cellular senescence [76,77] and the adoption of a senescent phenotype is thought to be a protective evolutionary mechanism rather than a direct driver of, or result of organismal aging. However, with increasing age, reduced efficiency of clearance of senescent cells can lead to accumulation and persistence of SASP [78]. This prolonged exposure to SASP causes detrimental effects on neighbouring cells through paracrine signalling, in turn triggering a cascade of premature cellular senescence as well as chronic inflammation and tissue degeneration [79].

**Figure 3:**
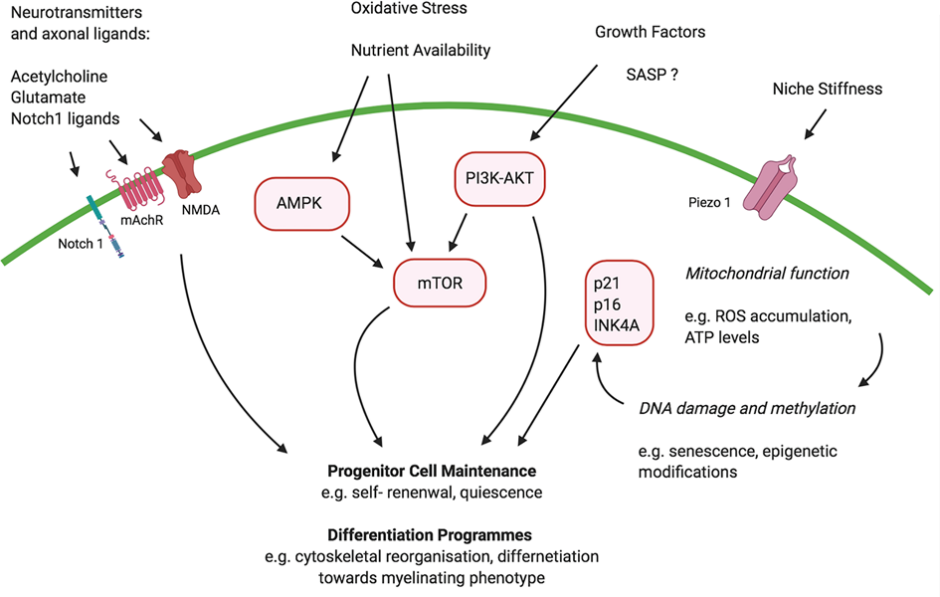
Hallmarks of cellular senescence in CNS cells Astrocytes, microglia and neurons have all been identified as accumulating hallmarks of cellular senescence including an increased expression of p16^INK4A^, p21 and SA-β-gal, senescence-associated heterochromatin foci (SAHF), telomere attrition and SASP production. It is not yet known if oligodendrocytes or their progenitors take on a senescent phenotype, although evidence of senescence-like features is accumulating. It is likely that their function is impaired indirectly by the accumulation of neighbouring senescent cells. Question mark indicates hypothetical involvement.

Hallmarks of cellular senescence such as increased expression of p16^INK4A^, p21 and senescence-associated β galactosidase (SA-β-gal) have been reported in astrocytes, microglia and neurons in the aging brain [80] (Figure 3). As well as reducing glial functions that promote myelination such as in efficient clearance of myelin debris, it is also possible that senescence could act on oligodendrocytes non-autonomously through SASP from these neighbouring senescent cell types in the CNS. For astrocytes and microglia, various SASP components, including inflammatory cytokines tumour necrosis factor-α (TNF-α), interleukin-1 β (IL-1β), interleukin-6 (IL-6) and SA-β-gal have been identified [81,82]. These cells appear to accumulate *in vivo*, and their clearance using senescence-reversing (‘senolytic’) compounds, which trigger apoptosis in cells of a senescent phenotype, appears beneficial to measures of cognitive function. A recent study found that in the PS19 mouse model of age-related τ pathology, there was an accumulation of astrocytes and microglia deemed to be of senescent phenotypes, due to p16^INK4A^ expression, which is associated with permanent cell cycle arrest. Clearance of these cells, using transgenic techniques and pharmacological intervention with senolytics, induced protection of cognitive function [83]. Although this was conducted in the context of neurodegenerative disease, it provides evidence that the occurrence of cellular senescence in glial cells may play a role in non-pathological, age-related cognitive decline.

Induction and accumulation of cellular senescence in neighbouring glia will affect the intricate cell–cell interactions which orchestrate and support oligodendrocyte function. Senescence in microglia impairs their ability to mediate immune responses, causes them to activate and release cytokines, pro-inflammatory factors and cytotoxic factors such as glutamate, aspartate and quinolinic acid and may therefore contribute to inflammation-mediated damage to oligodendrocyte cells. Due to their high metabolic activity, oligodendrocytes are particularly sensitive to the pro-inflammatory and cytotoxic secretions of activated microglia. Induction of microglial activation and inflammation in the rat brain, via injection of a bacterial foreign agent results in myelin degradation, measured by MBP expression, thought to be due to oligodendrocyte stress on exposure to inflammatory factors [84]. IL-1β and TNF-α, both released by activated microglia [85], are able to induce nitric oxide expression in oligodendrocytes, which has been linked to cell death and hypomyelination [86]. TNF-α induces apoptosis via direct mechanisms, binding to TNF p55 receptor, or via up-regulation of p53 [87,88].

Astrocytes have many roles in supporting oligodendrocyte function within the CNS, including homoeostatic control of metabolites, iron and cholesterol [89]. Cholesterol is an important factor in myelin synthesis [90]. Therefore, astrocytic senescence may contribute to reduced myelin production and a slowed rate of OPC differentiation [91].

Furthermore, senescent pericytes and endothelial cells are likely to compromise the permeability of the blood–brain barrier (BBB), which would have wide-reaching implications for the molecular and cellular environment which oligodendrocytes are exposed to in the aging brain. Pericytes form an essential interface with glial cells at the BBB and are implicated in many age-related diseases [92]. Grey matter pericyte degeneration has been associated with onset of cognitive dysfunction-associated diseases including AD and other dementias [93]. Pericyte-deficient mice show a premature loss of myelin from the corpus callosum, which precedes neuronal loss and results from hypoxia and accumulation of fibrin within the white matter. In fact, fibrin alone was able to simulate oligodendrocyte loss *in vitro* [93]. Therefore, with robust evidence emerging that other glia are vulnerable to cellular senescence, then the supportive roles these cells play in oligodendrocyte function will be reduced in the aging brain.

#### Disrupted axonal signalling

Axonal factors orchestrate myelination signals, for example by promoting trafficking of myelin proteins to the plasma membrane via activation of Notch1 signalling pathways [94–96]. Factors such as neuregulin-1 promote myelination, as overexpression of this factor causes hypermyelination *in vivo* [95]. The glial protein Fyn is thought to be important in integrating axonal signals within the oligodendrocyte. Neuronal adhesion molecule L1, which is released from axons, is able to activate Fyn kinase to enhance the local transcription of myelin proteins from mRNAs transported from the cell body [94,96,97]. Although oligodendrocytes can myelinate pseudo-axonal structures sufficiently *in vitro* in the absence of any axonal signals [98–100], it is clear that axonal cues including neuregulin-1 and L1 are able to influence the myelination process. Further, neuronal activity itself serves as an inducer of myelination [101–103].

Despite a negligible change in neuronal cell number in the CNS with age [104,105], there appears to be changes in cell properties. These include decreased soma size, reduced dendrite number and loss of dendritic spines, loss of synapses, altered neurotransmitter levels, altered response to neurotransmitters and altered electrophysiological properties [106–109]. Furthermore, the axolemmal connections at the paranode are weakened with age through loss of transverse bands [45]. Features of axonal degeneration are observed within the white matter in normal aging, including swelling of axons and elongation of mitochondria [110]. Age-related changes in the structure and function of neuronal mitochondria in white matter lead to increased vulnerability of aging white matter to free radicals and ischaemia, causing damage within the oligodendrocytes and their precursors, which are highly sensitive to oxidative stress [111]. This may be a mechanism by which age-related neuronal changes impact on myelin integrity.

Conversely, age-related myelin damage has been shown to trigger nerve dysfunction through secondary axonal changes. One of the major mechanisms for this is by paranode reorganisation, whereby myelin degeneration leads to loss of contact at the otherwise tightly anchored paranode region, resulting in disorganisation of ion channel distribution along the axon. Loss of clusters of ion channels at the nodes of Ranvier disrupts saltatory conduction [45,112]. Furthermore, this loss of interaction between the oligodendrocyte and the axon at the paranode seems to have profound effects on axonal integrity as knockout of specific myelin proteins proteolipid (PLP) and 2′,3′-cyclic nucleotide 3′-phosphodiesterase (CNP) alone can lead to paranode disorganisation and associated axonal degeneration [113]. This is thought to be due to loss of trafficking of RNAs from the soma to the paranode and associated axonal degeneration [114,115]. As discussed previously, neuronal activity alone acts as a cue for myelination, therefore it is possible that reduced nerve function due to myelin degeneration in turn may cause a positive feedback loop of reduced myelin maintenance or re-myelination. Further, myelin fragmentation, which has been widely reported in association with brain aging [116,117], leaves the underlying axon exposed and vulnerable to oxidative damage. Therefore, as discussed previously, it is unclear whether terminally differentiated neurons adopt a senescent phenotype with age, but nonetheless there are clear and numerous changes in axonal–oligodendrocyte signalling with age that are important to consider in the context of myelin maintenance.

### Intrinsic influences

#### Accumulation of DNA damage

As discussed above, the accumulation of senescent cells is thought to contribute to aging of tissues and organs. With the definition of cellular senescence as a termination of cellular division, this eliminates postmitotic terminally differentiated cells such as oligodendrocytes from this classification. However, the proposal has recently been made that the definition of cellular senescence should be broadened to include postmitotic cells [118]. Accumulating DNA damage is a characteristic associated with a senescent phenotype and may be indicative of ‘post-mitotic cellular senescence’ (PoMiCS). Although evidence for senescence programmes within postmitotic cells is limited, there are interesting observations which suggest that PoMiCS might offer a protective role in tissues such as the CNS, which largely consist of terminally differentiated cells, to avoid apoptosis of these specialised cell types in response to stress.

Oligodendrocytes are particularly vulnerable to oxidative damage due to their high metabolic needs [4]. It has been shown that oxidative-stress induced DNA damage can accumulate in oligodendrocytes with age [119]. This DNA damage indicates senescence as activation of the p53/p16 senescent pathway can be triggered by double-strand DNA breaks [80]. Accumulation of DNA damage, and a loss of function, specifically myelinating capabilities with age, form the basis of an argument for cellular senescence occurring in mature oligodendrocytes. Furthermore, oligodendrocytes are able to express a range of immunomodulatory molecules, including the SASP component, IL-1β, which protect from inflammatory damage [120]. However, the ongoing debate about the possibility of terminally differentiated cells undergoing senescence will likely continue until further characterisation is undertaken. There are currently no studies identifying SASP from oligodendrocyte cells, or other classical senescence hallmarks such as SA-β-gal. Considering the limited availability of senescence-specific markers and understanding of physiological roles of cellular senescence and its association with organismal aging, this warrants further investigation into the possibility of PoMiCS occurring within tissues such as the CNS. The development of senescence-specific biomarkers, implementation of animal models of accelerated aging and the effects of clearing senescent cells may advance our understanding in the near future.

#### Transcriptional changes

RNA sequencing studies have demonstrated that in the aging brain, there are significant alterations in transcriptomes of glial cells, compared with relatively little change in neurons. A large human brain study found that glial cell transcriptomes could be used as a robust indicator of brain age, and that oligodendrocyte-specific genes MBP and LINGO-1 (leucine-rich repeat and Ig-like domain-containing Nogo receptor interacting protein 1) were significantly down-regulated across all brain regions with increasing age [121]. A reduction in MBP is concurrent with a reduction in myelin content, as seen previously. However, LINGO-1 is a negative regulator of myelination. Inhibition of LINGO-1 promotes OPC differentiation via remodelling of actin filaments [122,123], which may also be significant for *de novo* myelination by mature oligodendrocytes. The gerontological mechanisms leading to these changes in expression are unclear, but it is feasible that they may contribute to a reduced capacity for oligodendrocytes to maintain optimal myelination via alterations in myelinogenesis or the remodelling of the actin cytoskeleton which is needed for active myelination [124].

Recent studies using careful transcriptomic analyses of mouse brains across lifespan have characterised changes in expression of key receptors and ion channels in OPCs with age. These include the G-protein coupled receptor Gpr17 [125], NMDA and kainite glutamate receptors [126], and voltage-gated sodium and potassium channels [126]. These are all important regulators of OPC excitability and differentiation, so age-related changes in expression towards an aged OPC phenotype correlate with decreased regenerative capabilities.

#### Mitochondrial dysfunction and oxidative stress

Oligodendrocytes have high metabolic requirements in order to maintain their specialised function in myelin synthesis and metabolic support of axons [127]. Mitochondrial dysfunction is therefore highly detrimental to myelin maintenance through reduced ATP levels, but it seems more importantly, due to the presence of reactive oxygen species (ROS). ROS can induce lipid peroxidation in myelin lipids, disrupting cellular homoeostasis and membrane integrity, which may contribute to age-related alterations in the structure and quality of myelin membranes. As mature oligodendrocytes appear to capable of maintaining their metabolic needs via glycolysis [128], which can occur within the myelin sheath itself [129], it seems that oxidative stress and accumulation of ROS-mediated damage may be more significant than energetic insufficiency in aged oligodendrocytes. Further investigation into mitochondrial function and oxidative status of mature oligodendrocytes, as well as changes in the expression of endogenous antioxidant mediators such as superoxide dismutase, catalase and glutathione peroxidase, is warranted to better understand the metabolic challenges facing aging oligodendrocytes.

## OPCs fail to sustain sufficient oligodendrogenesis due to intrinsic and extrinsic aging events

Despite net white matter loss with age, it is widely accepted that oligodendrocyte populations gradually increase in the adult cortex [42,130–133] and spinal cord [134]. Therefore, post-developmental oligodendrogenesis continues throughout life [134–137]. Post-developmental oligodendrogenesis is likely to be largely a result of OPC proliferation and differentiation, but oligodendrocytes can also derive from progeny of subventricular zone (SVZ) neural stem cells. With aging, the neurogenic capabilities of the SVZ declines, however this has been shown to not greatly affect the rate of oligodendrogenesis [138] and interestingly, with increasing age in the mouse, a higher proportion of SVZ-derived cells differentiate towards the oligodendrocyte lineage, shown by expression of NG2 [139]. In rodent studies, OPC density has shown no significant change with age [140,141]. However, the rate of OPC proliferation and differentiation does decrease somewhat with age [136,141], and the capability of OPCs to differentiate into mature oligodendrocytes with age may be region-dependent, such that dorsal OPCs have a reduced capacity to differentiate [142]. This may reflect different developmental origins of these progenitor cells. Therefore, OPCs retain the capacity to proliferate and to differentiate into mature oligodendrocytes throughout life, and although the rate of this oligodendrogenesis may be slowed with age, there is evidence to suggest that it does persist.

Recently, live imaging studies have shed some light on the fate of adult-born oligodendrocytes, using a transgenic mouse model that expresses fluorophores under the NG2 promoter. NG2 is a general marker of cells of oligodendrocytes lineage that is down- regulated as OPCs mature into oligodendrocytes. This enabled the repeat tracking of OPCs as they matured into oligodendrocytes, allowing fate-mapping of their path in real time [131,132]. These studies confirmed previous reports that oligodendrocyte numbers do increase throughout life, and that adult oligodendrogenesis contributed to formation of new myelin internodes. Interestingly, these studies also showed that mature oligodendrocyte phenotypes often only survived transiently and rarely integrated successfully, suggesting that the rate of production is much greater than the demand. Furthermore, zebrafish live imaging has shown that newly born oligodendrocytes had a window of 5 h to make myelin sheaths, after which very little alteration occurred with regard to the number and size of processes [143]. In agreement, studies on the human brain investigating carbon-14 integration into OPCs show that very few adult-born oligodendrocytes integrate into the CNS [57]. This suggests that although oligodendrogenesis occurs and has potential to contribute to re-myelination in the mature CNS, the scale of remodelling via this route is limited. However, it should be noted that factors such as increased neuronal activity and environmental enrichment can increase the rate of integration, by increasing OPC proliferation and differentiation [101,102,132]. Therefore, the physiological role of adult-born oligodendrocytes may be to alter conduction velocities by integrating new internodes, which could be a mechanism for learning and memory.

**Figure 4:**
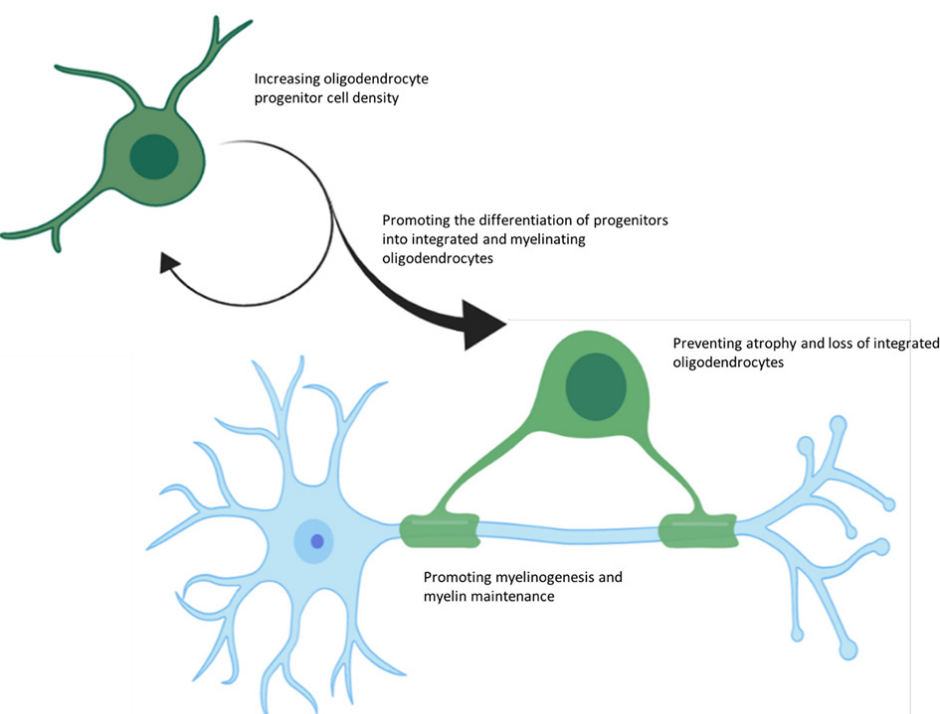
Possible signalling pathways implicated in OPC aging Key signalling pathways which underly the stem cell properties of OPCs include the AMPK, p13K-AKT and mTOR pathways. These integrate internal and external signals to regulate processes involved in proliferation and differentiation. In aging, signals regulating these pathways may be altered, leading to a reduced regenerative capacity. Abbreviation: AMPK, 5′-AMP activated protein kinase.

Studies of demyelinating diseases, such as multiple sclerosis, and artificially demyelinated animal models have revealed that it is a failure of OPC differentiation, as opposed to activation or proliferation, which underlies remyelination failure in response to white matter injury or demyelination [144]. The rate of this differentiation is also greatly impaired in the aging brain [145]. A reduction in differentiation efficiency is likely due to intrinsic changes within the OPC population as well as the result of an aging niche environment or altered signalling from other aging cells. These changes modulate key signalling pathways within OPCs which regulate their progenitor cell properties (Figure 4) [146]. Here, we will review some of these intrinsic and extrinsic factors which may contribute to a reduced regenerative efficacy of OPCs in the aging brain.

### Intrinsic influences

#### Cellular senescence

Evidence is emerging of senescence-like features accumulating in OPCs with age, which points to an intrinsic cellular aging program and are interesting to consider in the context of regenerative potential of these progenitor cells with age. OPCs display many features of stem cells, including self-renewal and multilineage potential [135,147]. Stem cells are capable of replicative and premature cellular senescence, and it has been suggested that cellular senescence in these cell types contributes to age-related disorders [75].

In culture, OPCs can continue to proliferate for extended periods, consistently for more than 6 months without showing signs of senescence, measured by SA-β-gal expression [148]. However, age-related expression of SA-β-gal and differences in proliferative capacity are important to consider. In contrast, *in vivo*, OPCs in the aged mouse brain show increased SA-β-gal expression [149]. OPCs isolated from aged rat brains show increased expression of senescence-associated gene *cdkn2a* as well as increased DNA damage, mitochondrial dysfunction and lower ATP levels, compared with those from young adults [150]. Recently, further hallmarks of senescence; up-regulation of p21, p16 as well as SA-β-gal, have been identified in OPCs isolated from human AD patients [151] and from demyelinated lesions of individuals with progressive MS [152]. Furthermore, these could be cleared using senolytic compounds quercetin and dasatinib, leading to alleviation of physiological and cognitive deficits associated with the AD model in mice [151]. Although these examples arise from cases of neurodegenerative disease, this suggests that OPCs can acquire a senescent phenotype in age-related disorders, which is associated with cognitive function, perhaps due to impaired remyelination as a result of inhibition of OPC differentiation. It would be of interest to explore this further within the non-pathological aging brain and study the effects of senescence on OPC switching from proliferation to differentiation.

### Epigenetic modifications

Age-related epigenetic modifications accumulate in OPCs which impair their differentiation capacity. It is likely that epigenetic rather than genetic alterations underlie the failure of OPC differentiation with aging, as it appears that OPCs in the aged mouse brain can be rejuvenated by exposure to a young systemic environment via parabiosis [153]. In this case, OPCs regained a young-like proliferative and differentiative response following focal demyelination, suggesting that the niche environment in the aged CNS is inhibitory to OPC function. It is possible that this is mediated via epigenetic modifications such as a reduced efficacy of recruitment of histone deacetylases [154,155]. Epigenetic changes, specifically age-related methylation appear to be modifiable via calorie restriction and fasting [156]. This is rapidly accumulating evidence that calorie restriction can reduce the aging phenotype in many tissues and organs, and even increase longevity [157]. Indeed, a recent study showed that OPCs are also sensitive to the effects of calorie restriction. Alternate day fasting in rats for a period of 6 months increased the regenerative capabilities of OPCs in response to a demyelinating lesion, and when isolated and cultures *in vitro*, these cells showed reduced hallmarks of cellular aging compared with freely fed control. These effects appeared to be mediated via modulation of the 5′-AMP activated protein kinase (AMPK) nutrient signalling pathway and could be replicated both *in vitro* and *in vivo* by use of the fasting mimetic, metformin [150]. Therefore, age-related changes in these nutrient sensing pathways are likely to contribute at least in part to the reduced regenerative capacity of OPCs with age, and modulation via dietary interventions or mimetics may reverse these epigenetic alterations.

### Extrinsic influences

As well as intrinsic cellular aging mechanisms which may reduce the capacity of OPCs to differentiate to produce myelin-forming oligodendrocytes, there is also age-related disruption of the extrinsic mechanisms which would normally orchestrate this process. The niche environment of OPCs confers cellular and molecular signals to the cells to drive their proliferative or differentiative behaviour. When these are altered in the aging brain, pro-regenerative capacity of OPCs is reduced.

#### Senescence in aging glial cells

As we have discussed above for mature oligodendrocytes, OPCs also rely on complex intracellular signalling and support, which mediates their function. Therefore, accumulation of cellular senescence in neighbouring glial cells can alter the environment of OPCs in the aging brain.

Myelin fragmentation increases with age, and fragments are largely phagocytosed by microglia [50,158]. Microglia appear to become less efficient with age, and the proportion of ‘dystrophic’ microglia increase in the human brain with age [159]. Given that the presence of myelin debris is inhibitory to myelination [160] and that the efficiency of clearance declines with age [161–163], the ability of oligodendrocytes to produce *de novo* internodes is reduced [117,164]. Furthermore, increased myelin fragmentation with age is thought to contribute to microglial senescence due to accumulation of the debris within the microglial cells [158,165]. Further evidence that aged glia influence OPCs in the aging brain comes from parabiosis studies. Infiltration of macrophages from a young mouse into a demyelinated lesion in the CNS of an older parabiotic partner was sufficient to increase OPC differentiation and remyelination via clearance of myelin debris within the lesion [153]. There is some evidence that OPCs themselves may also be capable of phagocytosis [10]. It will be of interest to investigate this phenomenon and its effects on debris clearance further in the aging human brain.

Pericytes are in close anatomical proximity of OPCs *in vivo* [166], and can directly influence OPC differentiation *in vitro* and *in vivo*. Further, re-myelination rates are slowed by a pericyte deficiency [167]. Therefore, there is good evidence that pericytes can stimulate the maintenance of a population of mature oligodendrocytes, providing another mechanism by which age-related pericyte degeneration may affect white matter integrity.

#### Peripheral aging

As parabiotic studies have elucidated, outlined above, exposure to a young systemic environment influences the behaviour and age phenotype of OPCs in the aged CNS [153]. While this seems to be in part due to the activity of other glial cells such as microglia, it is also important to consider the influence of systemic cellular and molecular components infiltrating the CNS. With age, the BBB becomes compromised, and hyperpermeable to various cells and molecules [168]. Significant accumulation of senescent cells has been reported in various tissues and organs with age. Therefore, the permeability to and influence of SASP and age-related circulating factors present in the systemic circulation warrants future investigation.

#### Axonal signalling and synaptic connections

Axons interact with OPCs in the CNS. OPCs can become depolarised and express various neurotransmitter receptors, including metabotropic and ionotropic glutamate receptors [169–171], and muscarinic receptors. Cholinergic signalling has been identified as a driver of OPC proliferation, preventing cell cycle exit towards differentiation [172]. The significance of this is not yet fully understood, but age-related changes in neuronal activity are likely to impact OPC activity via alterations in such mechanisms. In response to focal demyelination models in rodents in the cerebellar peduncle and corpus callosum, demyelinated axons generated new synapses with OPCs, and glutamatergic activation up-regulated OPC maturation [173,174]. Therefore, it is possible that axonal instability, as described previously, or reduced neuronal activity, will impair re-myelination due to reduced glutamatergic activation of OPCs. This may prevent re-modelling or repair within the aging brain. As well as neurotransmitter signalling, axonal signals, such as notch ligands interact with Notch1 receptors on OPCs which influence cellular pathways determining differentiation. The effects of Notch1 activation are dependent on age and have different roles in development and regeneration. Therefore, this might represent an interesting age-related mechanism by which axons affect OPC activity.

#### Niche stiffness

Recent work by Franklin and colleagues have uncovered further environmental influences which affect OPC behaviour in the aging brain, which are independent of cellular or molecular influence [145]. They have found that physical properties of the OPC niche alone are sufficient to determine their regenerative potential. Extracellular matrix within various regions of the brain, including the cortex, corpus callosum and striatum increase in stiffness with age. The mechanoreceptor, Piezo 1 which is expressed by OPCs detects this stiffness, resulting in decreased proliferative capacity of OPCs. Reducing the stiffness of the extracellular matrix, both *in vitro* and *in vivo* reversed the age-related reduction in levels of OPC proliferation. This provides novel insights into the extent of the variation of influences which affect cells of the oligodendrocyte lineage with age.

## Whole brain network connectivity and regional OPC subpopulations aging

Impaired cognitive function in aging has been attributed to a loss of network connectivity within and between brain regions [175]. A slowed processing speed is thought to be a major contributor to the reduction in executive cognitive function in aging [5,26]. Neuronal loss and damage, i.e. grey matter atrophy, has long been centralised as the focus for age-related deterioration of brain function. However, as discussed above, increasing molecular and cellular evidence of oligodendrocyte dysregulation in the aging CNS proposes a causal relationship between white matter loss and brain aging via the loss of essential roles of myelin in promoting brain connectivity via white matter tracts, as well as the multiple supportive roles myelin carries out in axonal metabolism, signalling and protection from oxidative damage previously described.

It is not yet well understood whether white matter degeneration precedes neuronal atrophy or *vice versa* in aging. Nonetheless, loss of brain white matter integrity not only disrupts gross connectivity, but also regional function. When considering white matter aging at the whole-brain level, it is also important to note that there is increasing evidence of anatomical heterogeneity in OPC phenotypes [126,176]. Although it is not yet understood exactly why these distinct phenotypes exist or their specific functional roles, it is likely that the difference in excitability and cell cycling affects the abilities of specific regions to maintain white matter differentially. For example, OPCs characterised by a high expression of NMDA receptors are largely found in white matter areas such as the cortex callosum while OPCs in largely grey matter areas have much lower expression [126]. This may reflect the ‘priming’ of OPCs in white matter regions to respond to activity-dependent activation. It is possible that with age, there is an increase in more quiescent phenotypes, reducing the overall plasticity of white matter in some regions more than others. Addressing these emerging questions within the field will provide a greater understanding of regional OPC phenotypes and potential interventions to promote ‘rejuvenation’ of quiescent OPCs to drive differentiation and myelination in aging.

## Interventions to protect white matter in aging

**Figure 5:**
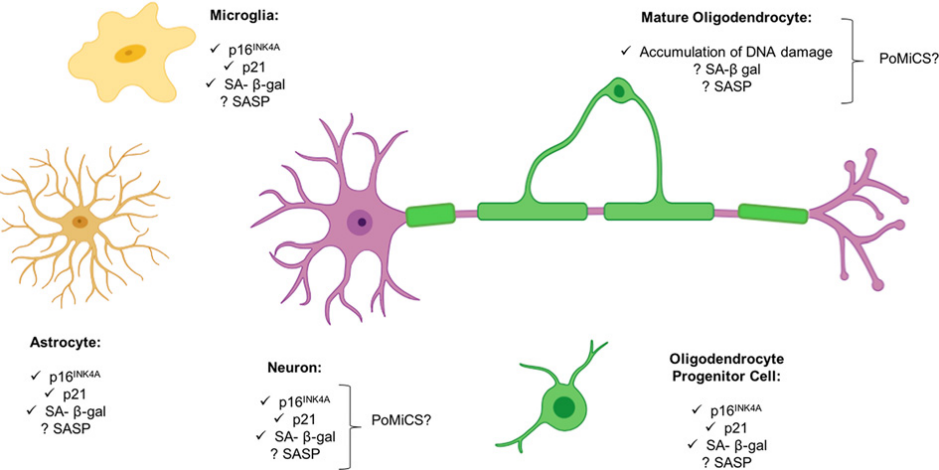
Strategies to protect oligodendrocytes and prevent white matter loss in aging Nutrient, pharmacological or lifestyle interventions may be able to support cells of the oligodendrocyte lineage to maintain high-quality myelin sheaths and prevent white matter loss with aging through various routes. They may increase the turnover and replacement of oligodendrocytes by OPCs by driving their proliferation and differentiation, or prevent oligodendrocyte loss through metabolic and antioxidant support, or support myelin synthesis by providing myelin precursors.

Interventions to support oligodendrocytes and myelin in the aging brain will protect against white matter degeneration and associated cognitive decline. Mechanisms of support will either promote oligodendrogenesis, and therefore increase potential for oligodendrocyte turnover through recruitment, differentiation and maturation of oligodendrocyte lineage cells from OPCs or neural stem cells or support the survival and synthesis of myelin by existing mature oligodendrocytes (Figure 5). The latter may be achieved by directly providing substrates for myelin membrane production and supporting metabolic demands, or indirectly protecting cells through anti-inflammatory and antioxidant mechanisms, or alternatively provide axonal protection which translates to myelination through pro-myelinating axo-glial signalling. OPC turnover and differentiation may be supported through rejuvenation of OPCs into a more active phenotype. As we have already discussed, white matter deterioration begins decades before the onset of age- related cognitive decline, it is likely that the most effective interventions will be preventative measures to protect white matter from mid-life, through specific targeting of oligodendrocyte lineage cells. Here we will briefly outline some of the recent findings which may provide potential therapeutic tools to support white matter through these mechanisms.

### Pharmacological

Cellular signalling pathways involved in the onset of developmental myelination are tightly controlled by direct axo-glial signalling and axonal secreted molecules. These include the Akt-1, Wnt, Erk and Notch signalling pathways, which are all involved in organising myelination patterns in the CNS, and promote the expression of myelin-associated genes [177,178]. These pathways provide potential therapeutic targets to promote myelin formation via pharmacological approaches. It is likely that during aging, this tight control is disrupted, perhaps due to preceding axonal degeneration, with implications on the maintenance of myelination. As alluded to already in this review, small molecule approaches to drive OPC differentiation are also emerging. Antimuscarinic compounds including clemastine fumarate and benzatropine have been shown both *in vitro* and *in vivo* to drive the differentiation of OPCs into mature and myelinating oligodendrocytes and improve white matter status following demyelination via interaction with OPC cholinergic receptors [179,180]. Another OPC receptor target is the Piezo 1 receptor, which is involved in mechanosensory signalling. Pharmacological inhibition of this receptor is able to mimic a more juvenile niche environment and rejuvenate OPCs *in vivo* [145]. As well as cell surface receptors, molecules to alter transcriptional regulation may also be beneficial to drive OPCs into a more differentiative state. For example, the mTOR signalling pathway is known to be a strong regulator of OPC differentiation, and the small molecule, LY294002, has been identified as a transcriptional regulator of this pathway with the ability to rejuvenate aged OPCs into a more primed phenotype [125].

### Nutritional

The process of myelin synthesis, membrane wrapping and maintenance is highly demanding on energy supply and nutrients [4]. Therefore, it follows that oligodendrocytes may have specific requirements in order to maintain their myelinating capacities. Nutrient approaches to support myelination could primarily provide direct substrates for myelin sheath formation as well as supporting energy supply and normal metabolic status of oligodendrocytes and modulating pathways involved in myelination and inhibition of differentiation. Myelin synthesis and maintenance requires sufficient delivery of proteins, lipids, fatty acids and micronutrients to the oligodendrocyte in order to synthesise the complex and large membrane structure. It is possible that this supply is compromised in aging. Evidence shows altered blood lipidome, including lower levels of myelin lipids such as sphingomyelin, is associated with reduced cognitive status in aging [47].

Beneficial effects of dietary interventions in demyelinating conditions such as MS and Pelizaeus–Merzbacher disease (PMD) include high-fat diet approaches, cholesterol supplementation and adherence to a ketogenic diet [181–184]. These are thought to act via anti-inflammatory mechanisms to reduce myelin damage, as well as via promotion of axo-glial pro-myelinating signalling. Furthermore, specific regional diets such as the Mediterranean diet, have long been understood to promote better cognitive outcomes in aging [185,186]. Therefore, there is accumulating evidence and interest that dietary interventions can modulate myelination within the mature CNS and may provide a protective therapeutic strategy.

Specialised nutrient mixtures as well as single nutrients have shown beneficial effects on white matter status following injury or demyelination. The multinutrient, Fortasyn® Connect contains the ω-3 fatty acids docosahexaenoic acid (DHA) and eicosapentaenoic acid (EPA), uridine monophosphate, choline and phospholipids, as well as vitamins B6, B12, C and E, folic acid and selenium [86]. Fortasyn® Connect was designed to support synapse formation in AD and shows some protection on cognitive function in mild AD [187], as well as neuroprotective effects in AD models [188], which have been attributed to neuroprotection via increased phospholipid metabolism [189]. More recently, this nutrient mixture has also been shown to protect against oligodendrocyte loss following white matter injury [190]. Similar nutrient mixtures or single nutrients may therefore be beneficial in supporting white matter in aging through provision of precursors and cofactors for myelin membrane synthesis. Many other nutrients have also shown beneficial effects on white matter protection in the context of damage or demyelination, including sphingomyelin [191], vitamins K and D [192–194] and taurine [195]. While the benefit of such nutrient support in the non-pathological aging brain remains hypothetical, there is strong evidence that nutritional interventions can be beneficial in the context of white matter repair following injury and demyelination, and in protecting cognitive function in pathologies associated with white matter, therefore their utility in promoting healthy aging through oligodendrocyte support warrants further research.

### Lifestyle

Beyond diet and nutrition, other lifestyle factors are able to promote oligodendrocyte health and myelination. Calorie restriction or fasting has shown interesting effects on OPC activity *in vivo*, with OPCs rejuvenating to a more proliferative state, followed by increased remyelination in a model of demyelination in aged rodents [150]. Finally, behaviours such as exercise and motor learning are other potential lifestyle interventions which may support white matter plasticity in aging through enhanced OPC differentiation and myelin remodelling by existing mature oligodendrocytes [63,184]. It is likely that future strategies to support white matter in aging will encapsulate a combination of nutritional, pharmacological and lifestyle factors to protect oligodendrocytes through support of myelination, protection from apoptosis and rejuvenation of OPCs.

## Concluding remarks

Here we have explored various intrinsic and extrinsic influences on both mature oligodendrocytes and OPCs which may contribute to the process of age-related white matter degeneration. To move our understanding forward on the state of oligodendrocytes in the aging process, a few key questions need to be answered. Firstly, the molecular and biochemical changes that occur in the aging oligodendrocyte are not well characterised and will provide insights into the mechanisms of myelin degradation. Secondly, a better understanding of the metabolic and nutritional needs of oligodendrocyte cells and how these change with age is needed to understand how to protect these cells into old age. Thirdly, an increased understanding and unanimous definition of cellular senescence as a mechanism for tissue aging, and the significance this may have in terminally differentiated cell types is important in understanding aging at a cellular level, and how the presence of senescent cells and their SASP may influence local tissue integrity. Fourthly, identification of specific biomarkers will shed further light on the role of senescence in brain aging. A growing research interest in use of senolytic compounds within the gerontology field may be of benefit to interrupt this positive feedback loop of senescence accumulation by enhancing the clearance efficiency of senescent cells and returning this cellular programme to one of regeneration rather than degeneration.

Aging is a highly complex and heterogeneous process, and white matter plasticity and integrity is influenced by many other factors such as diet, exercise and learning. This makes modelling age-related changes inherently difficult. Many studies on white matter plasticity and white matter changes in aging have been conducted in the rodent cortex due to experimental limitations of imaging deeper into the brain. However, as there are regional differences in white matter development, plasticity and function, it is likely that the cortex is not a suitable representative of the whole brain, especially of the deep white matter tracts involved in brain connectivity which are important in aging. Furthermore, age-related white matter loss in the rodent brain does not follow the same pattern as observed in the human and primate brains. For instance, the laboratory rat corpus callosum seems to be preserved from substantial age-related loss, while a smaller size of corpus callosum has been related to poorer cognitive abilities in aging humans [196], and myelination continues into old age. Therefore, the species and the brain region used for much white matter and aging research may not necessarily be directly translatable to the human condition. In addition, *in vitro* systems are useful for studying the process of sheath formation, however many of these rely on the production of myelin proteins as an indicator of increased myelination, although this is not the biological endpoint, it is not a true reliable index of myelin quality and functionality, since the process of integration and myelin wrapping is required. Therefore, future work is needed to model node and sheath formation, and to measure functionality, such as through assessment of conduction velocity.

In conclusion, white matter plays extraordinary roles in connecting brain regions and coordinating neuronal transmission, cognition, proprioception, motor coordination and sensory transmission. Although remarkably little research has focussed on the oligodendrocyte lineage in brain aging, great advancements have been made in recent years uncovering the intrinsic and extrinsic influences on oligodendrocyte function in the aging brain. White matter loss in aging is thought to be a major contributor to cognitive decline, leading to loss of independence in old age. Further work to understand the aging process of white matter holds potential to protect these functions through harnessing the adaptive and plastic nature of the oligodendrocyte lineage. While the many and diverse reasons for the loss in regenerative potential of white matter in age remain under investigation, it is clear that the CNS retains a high potential for myelin repair. This is supported by studies in multiple sclerosis, where extensive remyelination of lesions can be maintained even when patients survive into old age [197]. Overall, targeted therapies to support myelin maintenance in aging will maintain the important roles of white matter, particularly the connectivity within the brain that is necessary for optimal cognitive functioning into old age.

## Abbreviations

AD: Alzheimer’s disease
BBB: blood–brain barrier
CNS: central nervous system
DTI: diffusion tensor imaging
IL: interleukin
LINGO-1: leucine-rich repeat and immunoglobin-like domain-containing Nogo receptor interacting protein 1
MBP: myelin basic protein
MOG: myelin oligodendrocyte protein
NG2: neuron-glial antigen 2
OPC: oligodendrocyte progenitor cell
PoMiCS: post-mitotic cellular senescence
ROS: reactive oxygen species
SA-β-gal: senescence-associated β galactosidase
SASP: senescence-associated secretory phenotype
SVZ: subventricular zone
TNF-α: tumour necrosis factor-α
